# Analysis of Human Breath by Millimeter-Wave/Terahertz Spectroscopy

**DOI:** 10.3390/s19122719

**Published:** 2019-06-17

**Authors:** Nick Rothbart, Olaf Holz, Rembert Koczulla, Klaus Schmalz, Heinz-Wilhelm Hübers

**Affiliations:** 1 Institute of Optical Sensor Systems, German Aerospace Center (DLR), 12489 Berlin, Germany; heinz-wilhelm.huebers@dlr.de; 2 Department of Physics, Humboldt-Universität zu Berlin, 12489 Berlin, Germany; 3 Fraunhofer Institute for Toxicology and Experimental Medicine ITEM, Biomedical Research in Endstage and Obstructive Lung Disease Hannover (BREATH), 30625 Hannover, Germany; olaf.holz@item.fraunhofer.de; 4 The German Center for Lung Research (DZL), 35043 Marburg/35392 Giessen, Germany; rkoczulla@schoen-klinik.de; 5 Department of Pulmonology, Institute for Internal Medicine, Philipps-University of Marburg, 35043 Marburg, Germany; 6 Schön Klinik Berchtesgadener Land, Department for Pulmonology, Teaching Hospital of the Philipps-University, 35043 Marburg, Germany; 7 Teaching Department of the Paracelsus University Salzburg, 5020 Salzburg, Austria; 8 IHP—Leibniz-Institut für innovative Mikroelektronik, 15236 Frankfurt (Oder), Germany; schmalz@ihp-microelectronics.com

**Keywords:** breath analysis, millimeter-wave, terahertz, spectroscopy, molecular spectroscopy, gas sensing

## Abstract

Breath gas analysis is a promising tool for medical research and diagnosis. A particularly powerful technological approach is millimeter-wave/terahertz (mmW/THz) spectroscopy, because it is a very sensitive and highly selective technique. In addition, it offers the potential for compact and affordable sensing systems for wide use. In this work, we demonstrate the capability of a mmW/THz spectrometer for breath analysis. Samples from three volunteers and a sample from ambient air were analyzed with respect to 31 different molecular species. High-resolution absorption spectra were measured by scanning two absorption lines from each species. Out of the 31, a total of 21 species were detected. The results demonstrate the potential of mmW/THz spectroscopy for breath analysis.

## 1. Introduction

### 1.1. Breath Gas Analysis

The analysis of exhaled human breath is a very promising tool for medical applications. Since it is completely non-invasive, it has the potential to become a very convenient method for medical diagnoses or screenings. Furthermore, in contrast to a blood test, breath can be sampled fast and as often as required and an on-site measurement is possible. This allows also for the use of real-time studies.

More than 2000 years ago, Hippocrates started medical breath gas analysis when he realized that the aroma of breath can provide clues to diagnoses. Since then, physicians linked the smell of the breath to diseases, for instance the fruity odor of acetone to diabetes [1]. Nowadays, the alcohol breath test is a very prominent and well-established example for breath analysis. The blood alcohol concentration is approximated by the breath alcohol concentration, which is lower by a factor of roughly 2400 [2]. Compounds are exchanged between blood and breath by diffusion through the pulmonary alveolar membrane. However, the study of human exhaled breath can provide much more information than ethanol or acetone concentrations. Along with the main components nitrogen, oxygen, carbon dioxide, water, and inert gases, thousands of Volatile Organic Compounds (VOCs) were detected so far in human breath with a large variety between people and with concentrations ranging from ppt to ppm [3,4,5]. Some main VOCs common to all humans are isoprene, acetone, and methanol [3,6]. The compounds’ origins can be endogenous (from the host), from microorganisms (in lungs, mouth, and gut), or exogenous from the environment [7].

The large amount of information in breath offers a path towards a better understanding of metabolism and medical diagnosis. Reviews give an overview about the large variety of research involved with the analysis of human breath [7,8]. One example is the study of acetone concentrations in diabetes patients [9,10]. For patients with critical ketoacidotic diabetes, the acetone concentration can increase up to a few 100 ppm compared to around 0.5 ppm for healthy persons [11]. With more than 400 million diabetes patients worldwide, diabetes screening or monitoring has a large economic impact. Other human breath research includes studies on Chronic Obstructive Pulmonary Disease (COPD) or on systemic inflammation [12,13]. A method based on infrared spectroscopy is already successfully in use to plan liver surgery. With this method, the liver status of patients is determined by the exhaled isotopologue ratio of carbon monoxide after administration and metabolism of a ^13^C-labelled methacetin [14]. Breath analysis is also considered as a tool for therapeutic drug monitoring [15]. Due to its capability of fast and long-term sampling, real-time on-site measurements can be performed as well. Accordingly, it was observed that isoprene concentrations increase immediately with physical effort and acetone as well as isoprene levels change during sleep [4,16].

Sampling of the exhaled air is a crucial step in breath analysis. For instance, it has to be taken into account that the first portion of the exhalation is dead space, whereas the later portion contains alveolar air, which contains most of the valuable information [17]. Furthermore, external effects such as the ambient air, food and beverage intake, teeth brushing, smoking, and a number of further factors can influence the measurement and have to be considered [18,19]. Various methods for exhaled air sampling and storing, such as polymer/Tedlar bags, were demonstrated. Often, the sampled breath is pre-concentrated either by solid-phase microextraction or by thermal desorption [7,20,21]. Thermal desorption tubes filled with Tenax© are widely used and thus a good tool for standard sampling procedures. Standardization of sampling protocols is a major challenge in breath gas analysis and very important to compare different studies [19].

The method most widely used for the analysis of breath gas is gas-chromatography combined with mass-spectroscopy (GC-MS). This technology is very sensitive. However, it is not an option for regular clinical diagnosis because of the high cost of the instrument and very complex and time-consuming handling. Furthermore, light molecules with molecular masses below 35 g/mol, such as methanol, are challenging or not even possible to detect. Extensive overviews about breath analysis techniques are given elsewhere [11,22].

### 1.2. Millimeter-Wave/Terahertz Gas Spectroscopy

Millimeter-wave (mmW)/terahertz (THz) gas spectroscopy is based on rotational transitions of molecules which are excited by the radiation. Depending on the molecules’ structure, the strongest rotational transitions are typically located around 100–500 GHz [23]. For methanol, the strongest transition between 200 and 300 GHz has a line intensity of 8.3 × 10^−23^ cm^−1^/(molecule/cm^2^) resulting in 15% absorption at a pressure of 10 Pa per 1 meter absorption length [24]. As in infrared vibrational spectroscopy the mmW/THz spectra can be used for analytical purposes. A typical spectrometer for high-resolution mmW/THz spectroscopy consists of a radiation source, an absorption cell, a detector, and optical elements. The radiation is transmitted through an absorption cell, which is filled with a gas at a particular pressure and impinges on a detector, which generates an output voltage or current. The method is highly specific and selective due to the very high spectral resolution in combination with large spectral coverage. For example, a mmW/THz spectrometer with a transmitter and a heterodyne receiver can cover a frequency range of 100 GHz with a spectral resolution of 0.1 MHz. This results in 10^6^ spectral channels. Common breath gases, such as acetone, methanol, and ethanol, reveal more than 1,000 absorption lines within the 200–300 GHz range [24]. Furthermore, the absorption lines are as narrow as a few MHz at typical pressures of a few Pa, so there is little spectral overlap between the lines. Correspondingly, the spectra provide unique fingerprints for molecular species and the specificity and selectivity which can be obtained with a mmW/THz spectrometer can be considered as absolute [25].

MmW/THz gas spectrometers have been realized with different technologies and high sensitivity has been demonstrated [26,27,28,29,30,31,32,33]. Ethanol, methanol, and acetone have been unambiguously detected in human breath down to ppt levels [34,35,36]. In each of these works, the breath was collected in a Tedlar bag and pre-concentrators based on thermal desorption were used (custom-made [34] and commercial [35,36], respectively).

Due to its unique properties, mmW/THz spectroscopy can provide a valuable contribution to breath analysis. High sensitives that can compete with established methods were demonstrated. Furthermore, the specificity and selectivity is excellent such that mmW/THz spectroscopy can provide complementary information about molecules that can’t be detected by other approaches. Finally, mmW/THz spectroscopy systems are relatively simple compared to other techniques, enabling widespread applications in the future.

In this paper, we demonstrate sensitive and selective breath gas analysis of many different molecules by mmW/THz gas spectroscopy. The breath of three patients is sampled using a breath sampling device and Tenax© thermal desorption tubes.

## 2. Materials and Methods

### 2.1. Breath Sampling

Breath specimens were sampled using a setup at the Fraunhofer Institute for Toxicology and Experimental Medicine in Hannover. The sampling device and the sampling procedure were already established in previous studies using gas chromatography-mass spectroscopy for the breath analysis. A detailed description of the device can be found in Reference [37]. The volunteers breathed into an aluminum reservoir tube through a sterilized mouthpiece and a lung function sterile filter. By inhalation through a carbon filter, contamination of the inhaled air by VOCs in the ambient air was reduced. In order to get rid of residual contaminations, each volunteer breathed 3 minutes through the filter before sampling started. Subsequently, the persons continued breathing for 5 minutes while a constant airflow of 500 mL/min from the reservoir tube through the Tenax© thermal desorption pre-concentrator tubes was maintained by pumping. In total, 3 healthy male volunteers (P1, P2, P3) were sampled with P3 being a smoker (1 pack/day). In addition, one tube was loaded with ambient air during sampling of P1. The samples shall demonstrate the proof of our concept and were selected for practical reasons without claiming any valid representation. All samples were stored in the Tenax© tubes which were closed by a cap to keep them airtight. The tubes were carried to German Aerospace Center in Berlin, where they were measured on the next day.

### 2.2. Gas Spectroscopy Setup

**Figure 1:**
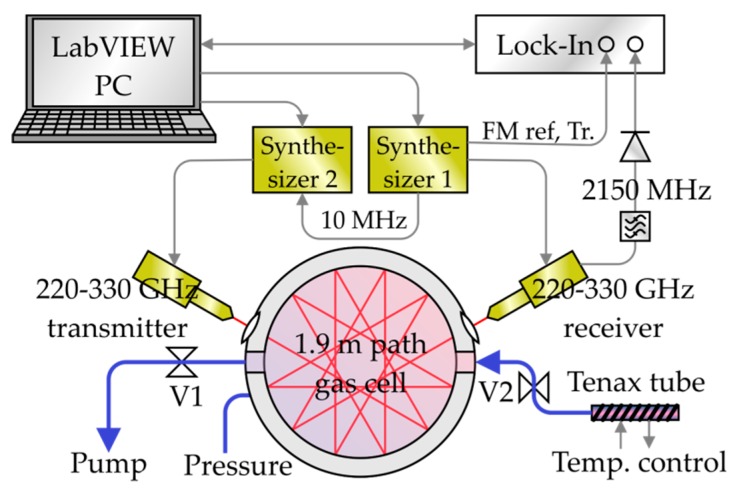
Setup of the mmW/THz gas spectrometer for breath analysis. The volunteer’s breath is released into the gas cell by heating of the Tenax© tube. The desorbed gas is analyzed by second harmonic (2f)-spectroscopy in the frequency range from 220 to 330 GHz.

The scheme of the mmW/THz spectrometer is shown in Figure 1. It is based on a transmitter (TX) and a heterodyne receiver (RX) (both from Virginia Diodes Inc., Charlottesville, VA, USA). TX and RX are equipped with diagonal horn antennas. They cover a frequency range from 220–330 GHz. The typical output power of the TX is −2 dBm. The RX has a 33 GHz IF bandwidth, a single sideband noise figure of 12 dB and a typical system conversion gain of +2 dB. A discussion of the sensitivity of a similar spectrometer can be found in [30].

The emitted TX frequency and the local oscillator frequency of the RX are controlled by two synthesizers operating between 9 and 14 GHz whose output frequencies are multiplied by a factor of 24. Both are synchronized to a common 10 MHz reference oscillator. A constant intermediate frequency (IF) of 2150 MHz between TX and RX is set. The IF signal at the output of the RX is filtered by a bandpass and rectified by a Schottky diode power detector (Agilent 8472B, Santa Clara, CA, USA). The TX is frequency-modulated with 30 kHz and a 500 kHz amplitude. The amplitude was chosen as a compromise between signal strengths and standing waves, both increasing with higher modulation amplitudes. The standing waves appeared as a result from a Fabry-Pérot resonance between the TX and the RX. A lock-in detector (Zurich Instruments HF2LI, Zurich, Switzerland) detects the second harmonic (2f) content of the IF power, which results in 2nd derivative-like shapes of the signals. This method enhances the sensitivity and reduces standing waves in the spectra. The lock-in amplifier is synchronized to the modulation frequency and trigger signals of synthesizer 1. The frequencies of the synthesizers and the lock-in amplifier are controlled with a custom-made LabVIEW program. A circular multi-pass gas cell with a total absorption length of 1.9 m is used. Two high-density polyethylene (HDPE) lenses focus the radiation from the TX into the cell and at the output of the cell into the RX. The gas cell has three vacuum ports for the pump (with valve V1), the pressure sensor, and the inlet. The Tenax© tube is connected to the absorption cell via a valve (V2). In order to facilitate thermal desorption the tube can be heated by a custom made heater based on a 50 Ohm resistor. The heater consists of an aluminum block where the tube is attached to. A temperature sensor placed close to the Tenax© tube and a temperature controller (Cryocon 24C, Rancho Santa Fe, CA, USA) allow for a precise control of the tube temperature.

### 2.3. Sample Handling and Measurement Procedure

In this section, the sample handling and measurement procedure is described. It includes the thermal desorption of the samples and inlet of the released gases in the gas cell as well as the measurement of the spectra. Each of the four samples, the ambient air and three volunteers’ samples, was handled the same way to allow for a comparison of the data. First of all, each Tenax© tube was connected to the heater and attached to the gas cell with the breath intake pointing towards the gas cell inlet. The other end was kept closed by the cap. The gas cell and the tube were evacuated to around 0.5–0.7 Pa (V2 open). The quite high pressure is due to outgassing of the Tenax© even without heating. Therefore, measurements were taken already at this pressure (run 0). Subsequently, valve V2 (cf. Figure 1) was closed while the pump remained evacuating the cell to a residual pressure of 0.01 to 0.1 Pa. The tube was heated to 250 °C, which took about 7 minutes, and stayed at this temperature for another 5 minutes before the released gas was let into the gas cell. This high temperature was chosen because it favors thermal desorption on the one hand. On the other hand, a high temperature might cause decomposition of some compounds, which has to be addressed in future work. With the valves V1 and V2, the pressure in the gas cell was set to 5 Pa. This pressure was chosen as an optimum between large signals and narrow linewidths. At this point, spectra of the breath sample inside the cell were taken (run 1). Individual absorption lines of 31 molecular species were scanned with a 10 MHz range around their center frequency. The species were chosen according to their relevance to breath analysis and the availability of spectral reference data. For each species, two absorption lines were scanned—except for carbon monoxide and hydrogen cyanide, each having only one absorption line in the available frequency range. The measured lines were chosen by their strengths and isolation from other lines. From the 31 species, 21 species were detected by at least one line in at least one sample. The detected species are shown in Table 1. The species that were not detected are ethylene, nitrogen dioxide, dimethyl ether, methyl chloride, acrylonitrile, propionitrile, sulfur dioxide, propionic acid, toluene, and ethyl benzene. This results from concentrations that are too low and/or from too weak absorption lines. After measuring the spectra, the gas cell was evacuated and filled again with 5 Pa from the heated Tenax© tube. With this filling, another measurement was performed in the same way. In total, each line was scanned four times, once before starting of the heating (run 0) and three times after the heating (run 1–3). Between the runs, there was a time span of about 20 minutes.

## 3. Results and Discussion

**Figure 2:**
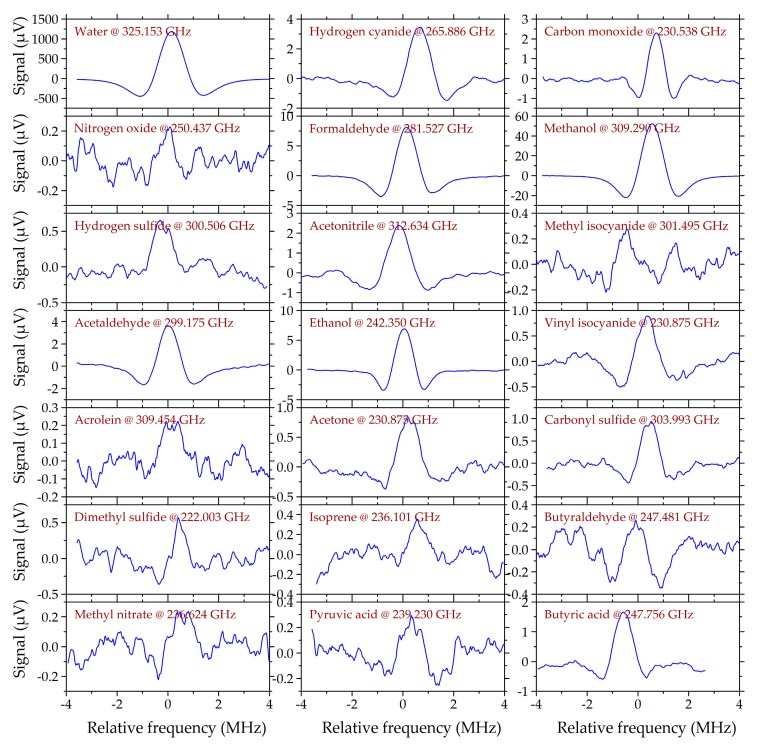
Line scans of the smoker’s sample (P3) with the largest signal for each molecular species. The signal-to-noise ratios range from 1 (acrolein, methyl isocyanide) to 6500 (water). The lock-in phase and the baseline of each scan were processed after the measurements.

Line scans with the largest signal for each species in the smoker’s sample P3 are shown in Figure 2. The derivative-like shapes result from the second harmonic (2f) detection, which is described in Reference [39]. The peak-to-peak signals vary by more than three orders of magnitude from 0.3 µV for acrolein and methyl isocyanide to 1630 µV for water. With the root-mean-square (RMS) noise level of 250 nV, this corresponds to signal-to-noise ratios between 1 and 6500. In a reference measurement of pure water vapor (line intensity 9.0 × 10^−23^ cm^−1^/(molecule/cm^2^) @ 325.153 GHz [24]) we observed a signal of 1870 µV. From that, we can estimate the water content in our sample to 88% and extrapolate the detection limit (SNR = 1) to 130 ppm without pre-concentration. Accordingly, the detection limit for hydrogen cyanide (line intensity 2.9 × 10^−20^ cm^−1^/(molecule/cm^2^) @ 265.886 GHz [24]) is approximately 400 ppb without pre-concentration. Each line was scanned in a range of 10 MHz around its center frequency with each scan taking 5 seconds at a 50 ms integration time and 100 Hz sampling rate. The modulation frequency was set to 30 kHz with a 500 kHz amplitude. For all scans, the phase of the lock-in amplifier was adjusted for maximum signal by post-processing of the data. The baseline was distorted by standing wave patterns with a 5 µV amplitude and 67 MHz period corresponding to a 2.2 m resonator which is the optical distance between TX and RX. The standing wave pattern was eliminated by post-processing of the data.

**Figure 3:**
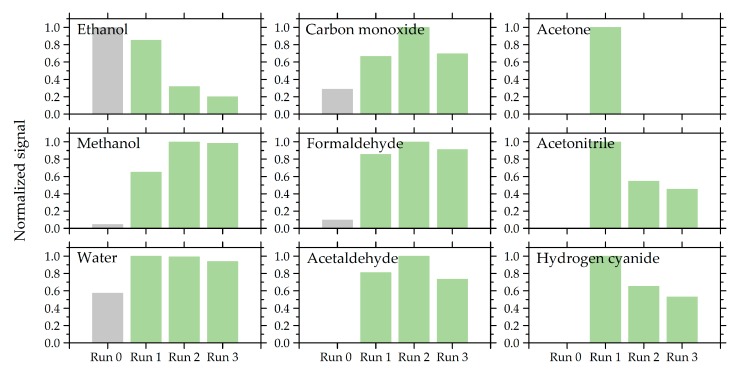
Variation of the amplitude between the subsequent scans of the P3 sample. Most species revealed the strongest signal in the first or second scan after heating (run 1 or run 2).

From all four scans for each species, the scan with the largest peak-to-peak signal was considered for the comparison between the volunteers. Most species revealed the largest signal in run 1 or run 2 after the heating of the tube started, meaning they were released easily, whereas some molecules were released later, as they provided the largest signal in run 3. But this signal was only slightly larger than that of run 2. Some examples of the P3 sample are shown in Figure 3. It should be noted that ethanol provided a large signal also in run 0 before we even started heating the tube. Therefore, the signal is not directly comparable with runs 1–3 measured at a pressure of 5 Pa. All other species were not detected or detected with much weaker signal in the scan before heating (run 0) than during heating of the tube (runs 1–3).

**Figure 4:**
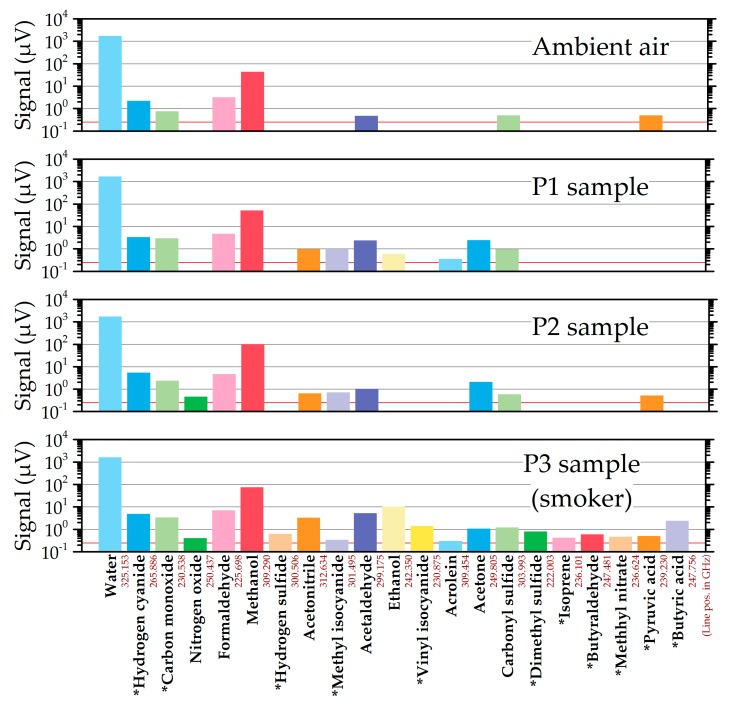
Results of the absorption line scans for ambient air and the three volunteers’ samples. For each detected species, the maximum peak-to-peak signal of the stronger absorption line is shown. Molecules, which were detected by only one transition, are marked by a star. The root-mean-square (RMS) noise level of 250 nV is indicated by the red line. The red numbers are the line positions in GHz.

In Figure 4, the maximum peak-to-peak signals measured for each species are shown for the ambient air and the three volunteers’ samples. Molecules marked with a star were detected by one line only. This might be a consequence of the second line scanned being too weak or of the fact that there is only one absorption line in the available frequency range (see Table 1). However, single line detection can in principle result from an unknown species; therefore the identification has to be considered individually and carefully in these cases.

It is important to note that the signal strengths given in the plot are not directly related to the concentrations of the species in the breath. For the determination of the concentrations in the volunteers’ breath, the concentration factors in the Tenax© adsorber have to be determined for each compound which can be done by reference measurements as described in Reference [20]. Furthermore, the signal strengths are directly related to the individual line strengths of each absorption line which are already cataloged for many molecules in data bases such as the JPL molecular spectroscopy data base [24], but not for all species so far. The data bases can be extended by reference measurements and quantum mechanical calculations. At this stage, our measurements allow only for a comparison between the samples from the patients as well as for qualitative statements. This is similar to several studies with other breath sensors as for instance published in [12].

The largest signals in all samples were measured for water and methanol. Comparably high concentrations of water and methanol are expected to be present in ambient, humid air and exhaled breath. However, despite quite some effort, contamination of the vacuum system, in particular from water, cannot be excluded because outgassing water is a general problem in vacuum systems. In all samples, hydrogen cyanide (HCN) was detected with signal strengths within a factor of 2.5 and with the lowest level in ambient air. It was detected with only one absorption line, because it is the only line in the available frequency range. However, the identification is very likely correct, because the measured line is very strong and HCN is a known component of atmospheric air [40]. It is furthermore known to have background levels of around 4 ppb in average in exhaled breath and has been suggested as a diagnostic tool for cyanide poisoning and for cyanide-producing bacterial infections [41]. Carbon monoxide (CO) was as well detected in all samples, but with three to four times higher signals in the volunteers’ samples than in the sample of ambient air. CO is a common pollutant in indoor and outdoor air having typical concentrations between 0.5 and 5 ppm [42]. Finally, formaldehyde, acetaldehyde, and carbonyl sulfide were detected in all specimens but with higher concentrations in the breath samples. These compounds were also detected in room air and exhaled breath in earlier studies [43]. Some species were detected in samples from all persons but not in ambient air, as for example acetonitrile and acetone. The latter is a breath gas common to all humans [3]. It was, for example, proposed as a biomarker for diabetes in case of very high concentrations [11]. Acetonitrile revealed a 3.5 to 5 times higher level in the smoker’s sample (P3). This result is in accordance with earlier studies, reporting higher levels of this component of tobacco smoke [44] in smokers’ exhaled breath [8,18]. Ethanol is present only in samples P1 and P3 with a 17 times higher level in the latter. Related to that, both persons revealed the highest levels of acetaldehyde (by factors of 2 and 5 compared to P2), which is a product of human ethanol metabolism.

Detections with only one observed absorption line include dimethyl sulfide or methyl nitrate. Dimethyl sulfide is a gas responsible for a pungent odor of the breath and it is linked to liver cirrhosis [45] whereas methyl nitrate was reported to be correlated with the acute, spontaneous hyperglycemia of type 1 diabetes mellitus of children [46]. In both cases, the detected signals were very weak such that the second line was possibly below the detection limit, because it has an even smaller line strength. For hydrogen sulfide, the second line at 314.4 GHz was particularly weak (by a factor of 4,000 compared to the first line at 300.5 GHz). Isoprene was also detected by only one very weak line and only in the sample of the smoker (P3), which is somewhat unexpected, because it is known to be a major constituent of breath for all humans [12,43]. Most likely, the isoprene transitions have very small line strengths. Unfortunately, the data base of isoprene is not complete and the line strengths are not known. Despite the excellent specificity of mmW/THz spectroscopy, single line detections have to be interpreted carefully and should be preferably confirmed by additional lines which were not found in our study.

Generally, it can be seen from the data that a large variety of compounds were detected in all samples with signals spanning three orders of magnitude. The volunteers’ samples revealed slightly more overall signals than the ambient air sample. In particular, the smoker’s sample resulted in the highest signals in most cases. In addition, this sample contained the highest number of compounds including several which were not detected in the other samples.

## 4. Conclusions

We have demonstrated sensitive and specific breath gas analysis by mmW/THz gas spectroscopy. This proof of concept approach was aimed to generate the first datasets on breathomics for three healthy volunteers among them one smoking healthy volunteer. Breath samples from the three volunteers along with one sample of ambient air were taken by an established sampling procedure with Tenax© adsorption tubes. The gas samples were thermally desorbed into a gas cell, where the gas mixture was analyzed with respect to a list of 31 molecular species. The molecules were identified by their mmW/THz absorption spectra. Two absorption lines of each compound (in two cases one line) were measured. In total, 21 different species were successfully detected in the samples. Until now the physiological and pathophysiological origins of the substances are not really understood but the sensitivity, specificity, and reproducibility of the measurement method allows for comparison between the samples. In order to increase the sensitivity of the system, future work will involve two measures. First, the sensitivity of the spectrometer will be increased by improving the absorption cell and the optics. Second, the gas extraction from the Tenax© tube will be improved, for example by purging and by reducing the water content. Furthermore it is desirable to quantify the gases’ concentrations in breath down to a level of ppt. For the quantification, reference spectra of some breath-relevant VOCs, as for instance isoprene, have to be measured in the mmW/THz frequency range. Including more absorption lines for each species and using versatile data analysis techniques, for example artificial intelligence methods, will improve the results. It has been shown that the analysis of complex mmW/THz molecular spectra benefits from such methods [47]. Another important step for the validation of the results is a systematic comparison with well-established methods such as GC-MS. However, even without the determination of absolute quantities, the results presented in this paper allow for a comparison between the individual samples, similar to previous breath analysis studies [12]. A particularly important development will be focused on the TX and RX. By replacing the current devices with TX and RX fabricated in CMOS or SiGe BiCMOS technology, the cost and complexity of the spectrometer can be drastically reduced, which may open the path towards widely used mmW/THz breath analysis.

It has been shown that the gas sensor approach has potential in VOC sampling. We aim to measure larger numbers of patients in well-defined groups in further studies. For medical purposes, the on-site technology and the straight approach of pattern recognition in VOCs is a promising technology.

## Figures and Tables

**Table 1:** Detected molecular species and corresponding scanned absorption lines sorted by the molecular masses M. The lines were chosen by strength and isolation from other known lines. The line positions were taken from [24] (M < 61) and [38] (M > 61). Lines marked with a star (*) were searched for but not detected in the spectra. Hydrogen cyanide and carbon monoxide have only one line in the spectral range of the spectrometer.

Compound	Molecular Formula	CAS Number	M (g/mol)	Line Positions (GHz)	
Water	H_2_O	7732-18-5	18.02	325.153	321.226
Hydrogen cyanide	HCN	74-90-8	27.03	265.886	-
Carbon monoxide	CO	630-08-0	28.01	230.538	-
Nitrogen oxide	NO	10102-43-9	30.01	250.437	257.822
Formaldehyde	CH_2_O	50-00-0	30.03	225.698	281.527
Methanol	CH_3_OH	67-56-1	32.04	309.29	241.7
Hydrogen sulfide	H_2_S	7783-06-4	34.08	300.506	314.438 *
Acetonitrile	CH_3_CN	75-05-8	41.05	312.634	239.119
Methyl isocyanide	CH_3_NC	593-75-9	41.05	301.495	301.461 *
Acetaldehyde	CH_3_CHO	75-07-0	44.05	299.175	312.784
Ethanol	CH_3_CH_2_OH	64-17-5	46.07	242.35	316.502
Vinyl isocyanide	CH_2_CHNC	14668-82-7	53.06	230.875	316.175*
Acrolein	CH_2_CHCHO	107-02-08	56.06	309.454	319.636
Acetone	CH_3_COCH_3_	67-64-1	58.08	249.805	316.224
Carbonyl sulfide	OCS	463-58-1	60.08	303.993	291.84
Dimethyl sulfide	CH_3_SCH_3_	75-18-3	62.13	222.003	256.269 *
Isoprene	CH_2_CCH_3_CHCH_2_	78-79-5	68.12	236.101	247.714 *
Butyraldehyde	CH_3_CH_2_CH_2_CHO	123-72-8	72.11	247.481	245.465 *
Methyl nitrate	CH_3_NO_3_	598-58-3	77.04	236.624	243.849 *
Pyruvic acid	C_3_H_4_O_3_	127-17-3	88.06	239.23	235.767 *
Butyric acid	CH_3_CH_2_CH_2_COOH	107-92-6	88.11	247.756	246.573 *
